# Focused Assessment with Sonography in Trauma and Abdominal Computed Tomography Utilization in Adult Trauma Patients: Trends over the Last Decade

**DOI:** 10.1155/2013/678380

**Published:** 2013-08-29

**Authors:** Alexander Y. Sheng, Peregrine Dalziel, Andrew S. Liteplo, Peter Fagenholz, Vicki E. Noble

**Affiliations:** ^1^Harvard Affiliated Emergency Medicine Residency, Massachusetts General Hospital, Boston, MA 02114, USA; ^2^Division of Emergency Ultrasound, Department of Emergency Medicine, Massachusetts General Hospital, Boston, MA 02114, USA; ^3^Division of Trauma, Emergency Surgery and Surgical Critical Care, Department of Surgery, Massachusetts General Hospital, Boston, MA 02114, USA

## Abstract

*Objective*. We sought to describe the trend in abdominal CT use in adult trauma patients after a point-of-care emergency ultrasound program was introduced. We hypothesized that abdominal CT use would decrease as FAST use increased. *Methods*. We performed a retrospective study of 19940 consecutive trauma patients over the age of 18 admitted to our level one trauma center from 2002 through 2011. Data was collected retrospectively and recorded in a trauma registry. We plotted the rate of FAST and abdominal CT utilization over time. Head CT was used as a surrogate for overall CT utilization rates during the study period. *Results*. Use of FAST increased by an average of 2.3% (95% CI 2.1 to 2.5, *P* < 0.01) while abdominal CT use decreased by the same rate annually. The percentage of patients who received FAST as the sole imaging modality for the abdomen rose from 2.0% to 21.9% while those who only received an abdominal CT dropped from 21.7% to 2.3%. *Conclusions*. Abdominal CT use in our cohort declined while FAST utilization grew in the last decade. The rising use of FAST may have played a role in the reduction of abdominal CT performed as decline in CT utilization appears contrary to overall trends.

## 1. Introduction

There has been a doubling of patient exposure to ionizing radiation in the last two decades in the United States. This increase is largely attributed to an explosive rise in the use of computed tomography (CT) [1]. Approximately, more than 62 million CT scans are currently obtained annually in the United States, compared to 3 million in 1980 [2]. Specifically, 18.3 million abdominal CT's are performed in the United States in 2007 [3]. Thus, in addition to increasing concerns about the rising cost of diagnostic imaging, there is growing and justifiable concern regarding health risks of radiation exposure [2–6]. Despite a frequently favorable benefit-to-risk ratio for the use of CT in symptomatic patients, increasing literature suggests an overuse of CT and questions its yield in specific contexts, including the evaluation of blunt trauma [7–12]. While much critique is based on pediatric data, the highest public health impact of reducing CT use may be in adults, specifically in reducing chest and abdominal scans in adult trauma patients aged 35–54 [3]. Thus, the Food and Drug Administration has recently proposed a national initiative to reduce radiation exposure from unnecessary imaging [13]. Concomitantly, the use of ultrasound in emergency medicine has blossomed [14–18]. Benefits of emergency bedside ultrasound include lack of radiation exposure, ability to perform imaging in the safety of the resuscitation room or in resource limited settings, and its ready repeatability as the patient's condition evolves.

Previous literature suggests that the establishment of an emergency ultrasound program, at least initially, may result in increased ordering of other diagnostic imaging, specifically formal consultative ultrasound [19–21]. However, as a program matures, the use of consultative ultrasound decreases dramatically [21]. In the intensive care setting, the use of bedside lung ultrasound has been shown to reduce the number of chest X-rays and chest CTs performed [22]. Nevertheless, definitive evidence correlating the use of ultrasound with the use of CT in trauma is lacking.

In our study, we sought to describe the trend in abdominal CT use in adult trauma patients before and after the introduction of a point-of-care emergency ultrasound program established at our hospital in 2004. Consequently, use of Focused Assessment with Sonography in Trauma (FAST) is now a routine at our institution. We hypothesized that there would be a rise in the number of FAST exams performed during our study period, and an inverse correlation between the number of FAST exams and the use of abdominal CT.

## 2. Materials and Methods

We performed a retrospective study of 19940 consecutive adult trauma patients age 18 or older admitted to our level one trauma center after ED evaluation from 2002 through 2011. Data were extracted from our trauma registry. Data points included age, gender, trauma mechanism, arrival time and date, length of stay in the emergency department, chief complaint, initial systolic blood pressure, Glasgow Coma Scale, head CT, FAST and abdominal CT results, injury severity score (ISS), [23] ED disposition, and mortality rates. Data was collected and entered into the trauma registry retrospectively through review of the medical record by trained research assistants blinded to the objectives of this study. FAST results were extracted from the emergency department physician and surgical consult documentation. Monitoring of our research assistance or assessment of inter-rater reliability amongst them was not performed. Our institutional Investigations Review Board approved the study.

FAST exams in our department were performed at the clinician's discretion without knowledge of our study and interpreted by emergency medicine and trauma surgery residents supervised by their respective attendings for the purpose of detecting intra-abdominal free fluid. A positive FAST was defined as the presence of any free fluid seen in the abdomen, most typically in the dependent areas of the peritoneum such as in Morison's Pouch, in the perisplenic space, beneath the diaphragm and/or in the rectovesicular recess. As we were interested in abdominal CT utilization, the cardiac view was considered irrelevant. A positive abdominal CT was defined by the trauma registry as hemoperitoneum, retroperitoneal, and pelvic hematoma, or any significant injury to abdominal organs or bowel. Isolated injury to bony structures was not included as a positive CT finding. In our cohort, the term “abdominal CT” was synonymous with abdominal and pelvic CT.

We plotted the percentage of patients in whom an abdominal CT and FAST were performed by year. We used head CT rate as control as a marker for overall CT utilization rates during the study period because as the use of decision support rules for head CT utilization in trauma patients became widespread, the rate of head CT ordering would be seen as stabilizing over this period. By contrast, there have been no such rules for abdominal or chest CT utilization in trauma patients, and therefore we anticipate a decline in head CT utilization rate over this time period relative to the rate of abdominal CT utilization. The trend of average FAST and abdominal CT rates per year over the study period was calculated by univariate regression. We also observed the percent of negative abdominal CTs during the study period.

All confidence intervals and *P* values were generated through statistical analysis using STATA (STATACorp, College Station, TX). We used Student's *t*-test for mean values of normally distributed continuous/quasicontinuous variables, ranked-sum (Mann-Whitney) test for non-normally distributed variables, and Pearson's Chi-squared test for percentage values to determine their statistical significance (*P* values). Multivariate logistic regression was used to control for changing clinical and demographic factors over time when assessing CT and FAST usage trends.

## 3. Results

19940 adult patients who were admitted to the trauma service during the study period were enrolled in our study. 474 (2.4%) of these patients were missing data on whether a FAST and/or abdominal CT was performed (187 were missing FAST data, 8 were missing abdominal CT data, and 279 were missing both) and therefore excluded from the analysis. Most (96%) of the excluded patients with missing data (453 out of 474 patients) were from 2002 because FAST and abdominal CT results were added to the registry after April, 2002. Demographic characteristics of patients with missing imaging data are outlined in Table 3. Thus, 19466 records with complete imaging data were included in the analysis. Of these, 11594 (59.6%) were male. The mechanism of injury was predominantly blunt (89.3%) rather than penetrating (10.5%).  Table 1 shows the patient demographics and the imaging performed per year. Patients who underwent FAST were on average younger (43.4 versus 59.0, *P* < 0.01), more likely to be male (70.9% versus 55.1%, *P* < 0.01), more likely to have higher median injury severity scores (ISS) (14 versus 9, *P* < 0.01), more likely to be admitted to the ICU (29.4% versus 9.0%, *P* < 0.01), more likely to go to the OR (19.1% versus 17.1%, *P* < 0.01), more likely to undergo abdominal CT (52.7% versus 11.7%, *P* < 0.01), more likely to have a positive result on abdominal CT (19.1% versus 12.3%, *P* < 0.01), and more likely to die (7.1% versus 4.2%, *P* < 0.01) when compared to patients who did not receive an FAST (Table 2).

In our study, 2904 patients received both FAST and abdominal CT. FAST had a sensitivity of 20.0% (CI 16.7–23.6%), specificity of 98.3% (CI 97.6–98.7%), positive likelihood ratio of 11.5, negative likelihood ratio of 0.81, PPV of 0.73, and NPV of 0.84 for predicting intra-abdominal injuries diagnosed on abdominal CT, the prevalence of which was 19.1% in this cohort.

**Figure 1:**
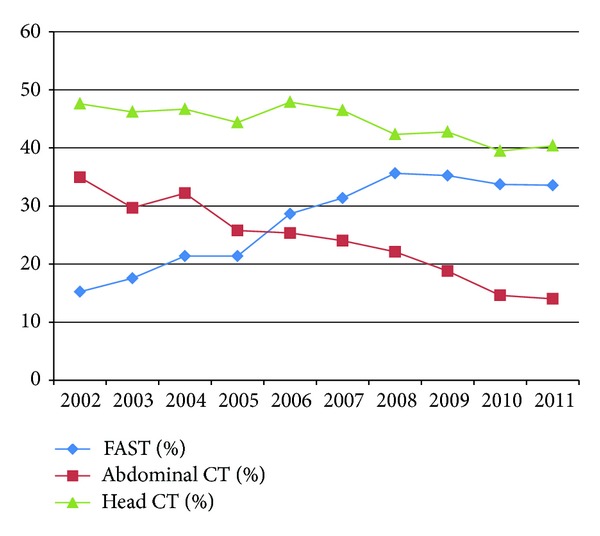
Percentage of patients in which a head CT, abdominal CT, and FAST were performed by year.

**Figure 2:**
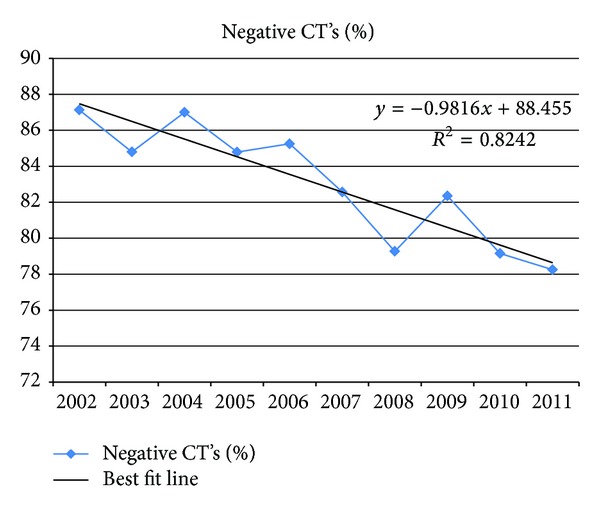
Percentage of patients in whom the result of the abdominal CT was negative by year.

**Figure 3:**
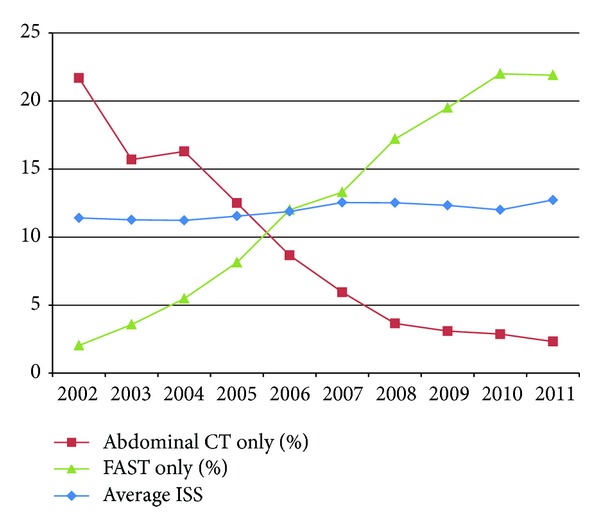
Percentage of patients who received only one imaging modality of the abdomen in the ED by year.

Overall, the use of FAST increased by an average of 2.3% (95% CI 2.1 to 2.5, *P* < 0.01) per year while abdominal CT use decreased by an average of 2.3% (95% CI −2.5 to −2.0, *P* < 0.01) per year (Figure 1). Head CT use decreased by an average of 0.8% (95% CI −1.1 to −0.6, *P* < 0.01) per year. If the head CT utilization rate is used as a surrogate for the overall trend of CT use independent of any potential impact of ultrasound, abdominal CT use still decreased by 1.5% annually. The percentage of patients in whom the result of abdominal CT was negative per year is shown in Figure 2. The percentage of patients who received FAST as the sole imaging modality for the abdomen during their trauma evaluation went from 2.0% to 21.9% while the percentage of patients who only received abdominal CT dropped from 21.7% to 2.3% over the last decade (Figure 3).

Almost half (2608 out of 5512 patients) who underwent FAST never required a CT at all. Of the 4539 patients who underwent abdominal CT, 2904 patients scanned with FAST were 6.8% less likely to have a negative CT result compared to 1635 patients in whom a FAST was not performed (80.9% versus 87.7%, *P* < 0.01). Those 2752 patients with a negative FAST who then underwent CT were 3.8% less likely to have a negative abdominal CT result, compared to 1635 patients in whom an FAST was not performed (83.9% versus 87.7%, *P* < 0.01). In effect, the FAST exam screened for those patients who were at increased probability for intra-abdominal injury. 

## 4. Discussion

Initial evaluation of trauma in the last two decades has seen a shifting paradigm regarding favored methods to screen for intra-abdominal injury. Diagnostic peritoneal lavage (DPL) has been largely replaced by FAST. At our institution, there has been an almost four-fold increase in the number of FAST performed over the last 10 years with a concomitant decrease in DPL use [24, 25].

Concurrently, overall CT utilization in general has grown tremendously in the evaluation of emergency department patients. The challenge has always been to discover ways to decrease CT utilization without compromising patient outcome. Abdominal CT boasts a sensitivity of 99%-100% for free fluid and can detect other injuries that do not result in free intraperitoneal fluid [26–31]. FAST, however, has demonstrated efficacy in detecting intraperitoneal fluid only and has not been shown to identify the source of free fluid or diagnose bowel or solid organ injuries. With increasing pressures to maximize value in healthcare and reduce radiation exposure, there has been growing interest in optimizing imaging utilization strategies. In our retrospective study, we sought to further explore the use of FAST by clinicians to identify the “minimally sick” and “maximally sick” in order to inform more efficient diagnostic imaging utilization.

Other studies have shown that ultrasound-based clinical pathways in the evaluation of blunt abdominal trauma reduces the number of CT scans from 56% to 26% without increased risk to the patient when the FAST is negative [32]. The same investigators estimated a cost saving of $450,000 at their institution by replacing DPL with ultrasound in their clinical pathway [32]. Another study described a two-third reduction in trauma care costs for patients who underwent FAST compared to those who underwent CT scan or DPL [33]. These studies suggest that physicians proficient in performing FAST have decreased CT utilization and greater diagnostic efficiency without increased incidence of missed injuries despite previously published test characteristics of FAST and those from our own data [34]. This suggests that despite FAST's known lack of sensitivity to rule out intra-abdominal injury, a certain cohort of patients can be managed using FAST without CT and that perhaps there are some injuries which are categorized as “missed” which may not be as clinically significant in terms of changing management or outcome. Better defining and standardizing which intra-abdominal injuries are essential to diagnose in the emergent trauma evaluation could help improve how FAST and other screening diagnostic tests are utilized. For instance, the clinical significance of a grade 1 liver laceration that requires no procedural intervention or change in disposition is still ardently debated [35]. Our study, which shows a correlation between increasing ultrasound use and a decrease in CT scan utilization for trauma patients, also suggests that FAST is being used as a stand-alone “rule out” test in certain cohort of patients. Until this practice is better understood and categorized, it is difficult to reconcile with published data.

Of note, the growth of FAST usage plateaued after 2008 which we hypothesized was due to providers having attained sufficient comfort level with its use in trauma after years of experience and the presence of a limit on the patients in whom FAST is considered clinically useful.

Interestingly, our results differed from the study by Inaba et al. that demonstrated a small but significant increase in CT utilization in trauma patients from 2002 to 2007; though the authors did not focus specifically on abdominal trauma and made no mention of ultrasound use [36]. Likewise, Roudsari et al. reported that abdominal CT use increased by 16% per year between 1996–2006 in patients over 55 years of age whose primary traumatic mechanism was a fall [37]. This suggests that as in our setting a mature ultrasound program may have a role in decreasing CT diagnostic imaging in low risk patients despite the low sensitivity that has been reported in the literature.

Moreover, ultrasound in our study was also associated with higher rates of CT utilization (52.7% versus 11.7% *P* < 0.01) in the patients with higher median ISS (14 versus 9, *P* < 0.01) and those who were more likely to be admitted to the ICU (29.4% versus 9.0% *P* < 0.01). This confirms our hypothesis that FAST is most useful in the extremes—in ruling in the sickest patients and focusing resources on them and in ruling out the least sick patients in whom injury is least suspected. Of the 4539 patients who underwent abdominal CT, 2904 patients scanned with FAST were 6.8% less likely to have a negative CT result compared to 1635 patients in whom a FAST was not performed (80.9% versus 87.7%, *P* < 0.01). Those 2752 patients with a negative FAST who then underwent CT were 3.8% less likely to have a negative abdominal CT result, compared to 1635 patients in whom a FAST was not performed (83.9% versus 87.7%, *P* < 0.01). This may demonstrate that a sicker cohort of patients undergoing FAST were screened by their providers and referred expeditiously to CT, which was higher yield after a FAST was performed.

Conversely, 2608 out of 5512 patients who underwent FAST never acquired a CT at all. Of these, 97 patients with a positive FAST who were hemodynamically unstable went directly to the OR. But for the vast majority (2461 patients), their negative FAST result appeared to have been sufficient to reassure the clinicians the absence of intra-abdominal injury. This is demonstrated by the fact that the percentage of patients who received FAST as the sole imaging modality for the abdomen during their trauma evaluation went from 2.0% to 21.9% while the percentage of patients who only received abdominal CT dropped from 21.7% to 2.3% over the last decade (Figure 3). This was surprising as the test characteristics for FAST were as noted above. This practice of “ruling out” intra-abdominal injury with FAST, which may be occurring with increasing frequency as ultrasound use became more prevalent, has been discouraged in the literature as the sensitivity of FAST, which confirmed to be low in our own cohort, has been demonstrated to be insufficient to rule out injury [38, 39].

But these studies were not done in trauma patients stratified by ISS. Patients with lower ISS may have a lower prevalence of clinically significant injury, and thus FAST scanning may be adequate to screen patients in such instances. This is an area that needs further study. Our results are consistent with the study by Branney et al. [32] which suggests that not only can FAST performance decrease diagnostic imaging but that it focuses resources on the management of the more severely injured patients. It also suggests, again, that diagnostic imaging strategies have to be developed in conjunction with mechanism of injury and ISS assessments and cannot be applied to “trauma patients” uniformly.

One point of interest from our analysis is that men were 15.8% (70.9% versus 55.1%, *P* < 0.01) more likely to undergo an FAST (*P* < 0.01) compared to women. We had initially attributed this to men having higher likelihood of severe injuries. While our data confirmed this, even after adjusting for ISS and ED disposition, men were still 11.1% (95% CI 9.9% to 12.4%, *P* < 0.01) more likely to receive an FAST. It is unclear why this may be the case.

The strengths of our study include the substantial study size (19466 patients). Nevertheless, our study has several limitations. The study is retrospective and as such we cannot establish a causal effect of FAST on CT scan utilization. Rather, the trends we identified are correlations. Like any retrospective study on ultrasound, the reliance on documentation for FAST results can be problematic; and no quality assurance efforts were made to review and confirm FAST results. However, of the 19940 adult patients included in the database, only 466 patients (2.3%) were missing data regarding whether a FAST was performed. Although most (96%) of the excluded patients with missing data (453 out of 474 patients) were from 2002 as FAST and abdominal CT results were added to the registry after April, 2002, we decided to retain data from 2002 in our analysis as we wanted at least 2 years of data before our point-of-care emergency ultrasound program was started to assess its impact. While other patient demographics remained similar during our study period, the median age increased by a decade; possibly due to an aging population as our trauma activation and admission protocols have not changed. Older patients underwent fewer abdominal CT's and FAST, likely due to the high instance of falls. For instance, 14.3% and 14.3% of >75 year-olds received an abdominal CT and FAST, respectively; while 28% and 33.6% of 35–55 year-olds received the same tests. As both FAST and CT were decreased as a function of age, the changing demographics over our study period likely affected FAST and CT equally. This was supported by univariate and multivariate logistic regression demonstrating that CT and FAST trends remained stable and statistically significant after controlling for age. We were unable to control for confounding factors such as changes in hospital system processes, ED and trauma attending practice patterns, and changing attitudes regarding the cost and radiation risk of CT that may have contributed to decreasing CT utilization. We attempted to mediate this limitation by using the head CT rate as a surrogate for the overall trend in CT use. While this practice has never been evaluated in other studies in the past, our choice to do so was based on the assumption that the Canadian Head CT Rules published in 2001 in theory should have impacted head CT utilization preferentially over abdominal CT use, for which evidence based practice guidelines are lacking [40]. Therefore, we believe that our findings are valid in that the trend to decrease abdominal CT utilization in our cohort is more marked. Our results differed from Lee et al., who reported a 60% increase in head CT use between 2003 and 2007 while the yield for positive results remained constant [41]. The difference is likely due to the fact that Lee's study reported head CT use in all patient populations while our study involved only admitted trauma patients. Because our database included only admitted trauma patients, we can make no comment on trends in FAST and abdominal CT use in discharged patients, for whom we were unable to gather data. While it is not the purpose of FAST to detect bowel or solid organ injuries, we noted an increasing trend in its use as a “rule out” test for intra-abdominal injury in selected low-risk patients. Although this has not been supported in the literature or by American College of Emergency Medicine clinical policies [42, 43], our aim was to simply describe this practice in hopes that future research can better delineate which population, if any, may benefit from an “FAST-only” algorithm without adverse outcomes.

## 5. Conclusions

From our analysis, we conclude that over the last decade in admitted adult trauma patients at our level 1 trauma center, the rate of FAST utilization has increased while abdominal CT use has declined. The rising use of FAST may have played a role in the reduction of abdominal CT use. Causation cannot be proven due to our inability to adjust for certain confounding factors that may have impacted the use of FAST and abdominal CT. Nevertheless, striking difference to the trends of increased CT utilization rates in the evaluation of emergency department patients in the United States is notable. Further study evaluating the impact of FAST results on the decision to order abdominal CT should be done, especially in patients at extreme ends of risk of intra-abdominal injury. Our study highlights the potential use of FAST as a screening diagnostic tool that could prevent unnecessary radiation exposure and minimize cost of care in a significant number of trauma patients.

## Figures and Tables

**Table 1:** Patient demographics and imaging studies performed by year.

Year	Number of patients	Median age (years)	Mortality (%)	Median ISS	Dispo to OR (%)	Dispo to ICU (%)	Number of FAST	Percentage of FAST*	Number of abdominal CT	Percentage of abdominal CT*	Number of head CT	Percentage of head CT*
2002	1279	49.2	6.25	9	2.22	1.48	195	15.25	447	34.95	609	47.62
2003	1792	51.5	5.80	9	2.29	1.46	315	17.58	532	29.69	828	46.21
2004	1791	49.3	4.58	9	2.24	1.53	383	21.38	577	32.22	836	46.68
2005	1857	51.5	5.22	9	1.78	1.46	397	21.38	479	25.79	824	44.37
2006	1940	53.6	4.54	9	1.80	1.38	556	28.66	492	25.36	929	47.89
2007	2072	53.1	4.92	9	1.56	1.43	650	31.37	498	24.03	963	46.48
2008	2110	54.4	4.69	9	1.64	1.48	752	35.64	467	22.13	893	42.32
2009	2168	55.1	5.30	9	1.54	1.47	764	35.24	408	18.82	927	42.76
2010	2262	56.1	5.00	9	1.58	1.50	763	33.73	331	14.63	893	39.48
2011	2195	59.0	4.46	9	1.41	1.57	737	33.58	308	14.03	886	40.36

*Percentage of total patients who received the listed imaging test during their trauma evaluation in the ED.

**Table 2:** Comparison of demographics between patients who received an FAST and patients who did not.

	FAST not performed (*N* = 13954)	FAST performed (*N* = 5512)	All patients (*N* = 19466)	*P* value
Median age (years)	59.0	43.4	53.7	<0.01
Male gender (%)	55.1	70.9	59.6	<0.01
Median ISS	9	14	9	<0.01
Admitted to OR (%)	17.1	19.1	17.7	<0.01
Admitted to ICU (%)	9.0	29.4	14.8	<0.01
Mortality (%)	4.21	7.08	5.02	<0.01
Abd CT performed (%)	11.7	52.7	23.3	<0.01
Abd CT positive (%)	12.3	19.1	16.6	<0.01

**Table 3:** Comparison of demographics characteristics between patients with complete imaging data and excluded patients.

	Included patients	Excluded patients	Entire cohort
Number of patients	19466	474	19940
Median age (years)	53.7	46.0	53.5
Gender (% male)	59.6	60.5	59.6
Median ISS	9	9	9
Admitted to OR (%)	17.7	16.5	17.7
Admitted to ICU (%)	14.8	13.5	14.7
Mortality (%)	5.0	6.5	5.1
